# Arts, place, and sacrifice zones: restoration of damaged relational values in a Chilean sacrifice zone

**DOI:** 10.1007/s11625-022-01252-6

**Published:** 2022-12-15

**Authors:** Teresa Sanz, Beatriz Rodríguez-Labajos

**Affiliations:** 1 Institute of Environmental Science and Technology, Universitat Autònoma de Barcelona, Campus de la UAB, 08193 Cerdanyola del Vallès, Barcelona Spain; 2 Johns Hopkins University-Universitat Pompeu Fabra Public Policy Center, Universitat Pompeu Fabra (UPF), 08002 Barcelona, Spain; 3 UPF Barcelona School of Management, Barcelona, Spain; 4 Energy and Resources Group, University of California, Berkeley, USA

**Keywords:** Sacrifice zone, Relational values, Artistic practices, Restoration, Placemaking, Socio-environmental conflicts, Chile

## Abstract

This paper aims to unpack the relational dimension of place and placemaking by analysing how creative actions underpin relational values towards socio-spatial restoration in the sacrifice zone affecting the communities of Quintero and Puchuncaví (QPSZ) in Chile. Sacrifice zones are places permanently subject to environmental damage and lack of environmental regulation. For affected populations in environmentally degraded areas, creative actions such as murals, music, and street performances have become a way to re-establish connections both among humans, and between humans and the environment. To date, little has been theorized on this connection. With this in mind, we use network analysis to analyze which and how relational values are mobilized by artistic actions, and to examine ensuing socio-spatial transformations. Drawing insights from 35 interviews with activists, artists, and residents in QPSZ, we observed relational effects of arts, especially in creation processes, and in representations of local elements and life histories. The materiality of artistic practices raised as a force of placemaking, and so did artistic spaces as promoters of networking and social cohesion, essential for socio-spatial restoration. By bringing together insights from aesthetic politics, human geography, and relational values, this paper contributes to the emerging literature on art committed to tackling socio-environmental crises, and to political–ecological theories on the transformation of degraded areas.

**Supplementary Information:**

The online version contains supplementary material available at 10.1007/s11625-022-01252-6.

## Introduction

While global leaders debate whether or not to end coal investments (Harvey 2021) to meet the target of the 2015 Paris Agreement (UNCCC 2015), coal-related industries keep turning many places around the world into sacrifice zones (Lerner 2010). A sacrifice zone is a term used to characterize a geographic area that has been permanently subject to environmental damage and lack of environmental regularization (Bolados 2016). In Latin America, most of the sacrifice zones derive from a development model based on neoliberal policies from the 1980s; the resulting emphasis on the economy and economic dependence on external markets caused degradation of the region’s natural commons (Espinoza 2016; Montaña et al. 2005; Svampa 2012). Such development-based governance obscured the environmental impacts and land degradation, on the one hand, and the damage to local communities’ material, symbolic, and spiritual lives (Gudynas 2013; Svampa 2019), on the other.

In this sense, neoliberal processes are arguably sustained by the promotion of privatization, alienation, and individualization throughout all political formations, interconnections and networks (Bridge et al. 2013; Peck 2010). As a result, relational values subside. Relational values are meaningful human–environment connections and social relations (Riechers et al. 2020) that represent the “ecological, cultural, and social interdependence” of identities and communities (Muraca 2016: 31). This term was proposed to widen intrinsic and instrumental values associated with nature and include the values derived *from* human–ecosystem relationships (Chan et al. 2016), thus values that are relational in content (e.g. stewardship, care, cultural identity, etc.) (Himes and Muraca 2018). Critical social-spatial theory points to the role of social interaction for placemaking to identify the physical, social, and experiential fragments that constitute places (Escobar 2001; Lefebvre 1991; Massey 2005; Nicholls 2009; Pierce et al. 2011). Artistic practices have been recognized as relational and a constitutor of connections among humans and between humans and the space (Hawkins et al. 2015; Dixon 2009; Whatmore 2006), especially in the context of community fracture (Hawkins and Straughan 2015; Yusoff et al. 2012). Nevertheless, a gap persists on how the arts challenge the material and symbolic configuration of sacrifice zones through the activation of relational values.

In response, this paper takes recent attempts to develop relational values for landscape restoration forward, including how territorial and community identities, social cohesion, and cultural practices are mediated by the state of the environment (Chapman et al. 2019; Riechers et al. 2019). Our intent is to unpack the connection between artistic activism, relational values, and sociospatial transformations in environmentally degraded areas. Thus, this paper aims to contribute to geographers’ theorization of the relationality of art (Dixon 2009; Hawkins et al. 2015; Askins and Pain 2011) and place (Escobar 2001; Massey 2005; Pierce et al. 2011), by exploring the role of artistic activism in placemaking. We explore the artistic expressions related to the Quintero–Puchuncaví sacrifice zone (QPSZ) in Chile, and categorize the relational values connected to art practice. Then, we analyse how artistic actions enhance damaged relational values, through representation and through experience, and how this enhancement transforms the social and spatial dynamics of place. Our analysis engages with literatures on contemporary art theory, human geography, and relational values, and thus contributes to the study of art committed to the socio-environmental crisis, and to political–ecological theorizations of the transformation of degraded areas.

The remainder of this paper is divided as follows. The next section offers a literature review about sacrifice zones, the crisis of relational values, and the relationality of art. After presenting the research methods, the paper overviews the analysed creative actions, and presents the relational values fostered by artistic experience and representation in the case under analysis. The next section explores transformation trends associated to the described processes. The final section connects the theorization of relational aspects of artistic practices, place, and the socio-environmental restoration of degraded landscapes.

## Sacrifice zones and crisis of relational values: introducing the sacrifice zone Quintero–Puchuncaví

A sacrifice zone is a geographic area where industrial development has devastated the environment to the impairment of fundamental rights of the residents (typically low-income populations), including rights to life, health, education, work, food, and housing (TERRAM 2019). In these places, pollution has not only impacted the water and soils that sustain traditional economies (e.g. fishing and agriculture) but also devalues the local history, identity and practices that were co-created with the space (Fernández-Llamazares et al. 2022). Local governments, civil society organizations, and grassroots groups contest this model of production and land use because of its socio-environmental impacts and object to its lack of recognition of alternative lifestyles (Svampa and Viale 2014; Valenzuela-Fuentes et al. 2021). A clear example is the silencing of indigenous and local communities, and their values, knowledges, and ways of being by production focused decisions of power groups (Martínez Alier 2007).

The predation of history, culture, and traditions by industrial activities is such that sacrifice zones become what Augé (1993) defined as “non-places” (Hormazábal et al. 2020), that is, locations that cannot be defined as historical or relational spaces or spaces of identity. For Augé, *place* is a space of identity, affectivities, traditions, and histories dependent to the space, both materially and symbolically. This aligns with Doreen Massey’s theory on the central role of relations, embedded in material practices, in the construction of place (Massey 2005). Such relations have important values associated to them, a point that environmental scholars stress to counter discourses on the commodification of nature and the instrumentalization of its services (Gudynas and Acosta 2009; Temper and Martinez-Alier 2013).

Attempts to analyse the relational values eroded by landscape simplification (Hanaček et al. 2021; Kaltenborn et al. 2017; Riechers et al. 2020) and social analyses of sacrifice zones (Hormazábal et al. 2020; Hormazabal et al. 2019) typically focus on cultural identity, social cohesion, and sense of place. In general, these approaches indicate that overcoming value erosion entails the embodiment of new identities and care. Authors analysing relational values acknowledge that activities such as fishing, gardening, and bird-watching, as well as group activities that involve playing, celebrating, and struggling together convey care, and create options to know the place and other inhabitants (Chan et al. 2016). Artistic practices connect with identity (Jenkins 2010; Serafini 2018), care (Barros 2020) local knowledge (Rodriguez-Labajos et al. 2021), and agency (Molina Barea 2018). Yet the scientific literature lacks contributions that address how engaged arts can be the pathway to restore the degraded landscape through enhanced relational values.

In Chile, like other export-oriented economies, the spatial transformations led by foreign capital, resulted in spatial and socio-cultural sacrifices (Bolados 2016). In 2019, former President Sebastian Piñera promised to end the dependence on coal by 2050 (DW 2019). Nevertheless, 28 coal-fired power plants still operate throughout the country. All but one operate in the five heavily environmentally impaired areas known as “sacrifice zones”. After 1980, neoliberal policies started a territorial articulation around industrial development and exploitation of Chilean natural resources. Polluting industries clustered in rural areas without a regulation by state authorities (Espinoza et al. 2016), giving way to intense environmental degradation. The area selected for this research is a clear example.

The QPSZ is one of the biggest industrial complexes of Chile, pivotal to the development of the national economy (Espinoza 2016). The industrial park hosts more than seventeen industrial facilities, including power plants, petrochemical and oil refining industries, chemical processing companies, gas terminals, and a copper processing plant (EJAtlas 2020). The region of Quintero–Puchuncaví, as other sacrifice zones, has turned into a non-place. Local residents started suffering health issues presumably caused by industrial pollution (Navas et al. 2022) and the loss of livelihood and traditional practices eroded sense of place and local identity (Tironi and Rodríguez-Giralt 2017). In this context of unceasing impacts of pollution and degradation on the collective identity, scholars acknowledge the emergence of new identities ranging between everyday suffering and resistance (Hormazábal et al. 2020; Valenzuela-Fuentes et al. 2021). In parallel, *care*—expressed both as self-care and as mutual support—becomes a mode of “knowing and acting upon their harmed bodies and environments” (Tironi and Rodríguez-Giralt 2017: 92). This case offers the opportunity (and the urgent need) to analyse the impact of resistance identities and practices in the re-configuration of the public space.

## Artistic actions and relational values

To explore the relationality of artistic practices, this paper explores two components. The first is art’s *representation* potential, that is, the *content* of the artwork that is expressed, and how the artwork expresses it. The second is the spaces of *experience* of the art producers, especially in community arts, through the production and reproduction of certain dynamics and values among the participants in the space.

### Artistic representations and place

Artistic actions have historically expressed humans’ diverse meanings of nature. For instance, the paintings by Caspar David Friedrich or J. M. W. Turner expressed “nature” as a divine creation against the artifice of civilization—in line with the Western Environmentalism’s notions of “nature” as something separated, distinct, and independent from humans (Muraca 2016). Outside the museums and professional artist circles, local environmental movements and communities around the world use their creativity to express their discontent over projects and to promote environmental transformations (Sanz and Rodriguez-Labajos 2021). These paintings, banners, street performances, and songs directly express people’s relationship with the environment (Belalcazar Valencia and Molina Valencia 2017; Jenkins 2010; Merlinsky and Serafini 2021; Ogaga 2011). In other words, local communities use certain representations (i.e. images, icons, words) in their artworks to express their meanings of place.

Scholars addressing relational values acknowledge the meanings imbedded in certain cultural elements. Trees (Chan et al. 2016; Eriksen and Ballard 2020) and regional food (Riechers et al. 2021) represent community values such as local identity. In Hawaiian culture, certain concepts referring to human–ecosystem relationships embody notions of balance, care, and responsibility (Gould et al. 2021). Likewise, arts contribute to repairing the “social bond” (Dixon 2009: 412) by re-defining notions about the commons.

Dixon (2009) draws on Jacques Rancière’s work (2002) to argue that artistic representations of biodiversity challenge the current configuration of politics, focusing on the idea that artistic practices “reframe the network of relationships between spaces and times, subjects and objects, the common and the singular” [2009: 412]. Community arts against fracking in Argentina used paintings to represent the material processes behind the environmental struggle, thus shaping “symbolic, relational and material forms of cultural resistance” (Serafini 2018: 10). Again, through the lens of Rancière, aesthetics is political as it has a role in shaping the collective worldview.

Critical thinkers acknowledge the relational nature of place as symbolically constructed (Escobar 2001; Lefebvre 1991; Massey 2005; Nicholls 2009; Pierce et al. 2011). For instance, Lefebvre’s classification of the space describes “spaces of representation” as all the mental inventions (including codes, sign discourses, imaginary landscapes, symbolic spaces, paintings) that confer new meanings to spatial practices (Lefebvre 1991).

In turn, the new meanings constructed by acts of imagination operate as material forces in the production of place, as Harvey (1989) notes referring to the political, social, and economic action that aesthetics and arts motivate. However, human geography theories confine themselves to acknowledging artistic expressions as sources of information about communities’ meanings of place, without elaborating on their role in placemaking. In particular, there is little attention to the way artistic representations of place enhance alternative human–environment relations in places commodified by extractive projects. For example, in Southern Chile, the Mapuche–Pewenche indigenous people’s culture, history and spirituality is based on a reciprocal relation with the Pewen (*Araucaria araucana* trees) (Ibarra et al. 2022). There are artists, e.g. Leonel Lienlaf (1989) (see Ibarra et al. 2022) and Cecilia Vicuña (see Barros 2019), that represent and enhance through poems and visual arts these alternative worldviews that have been historically silenced through colonialism and stereotypical images of indigenous identity (Contreras 2021). Therefore, from an environmental justice perspective, representation is critical to overcome the misrecognition of identities that challenge the hegemonic model of production (Bolados 2016; Bourdieu 1991; Escobar 2001; Kojola and Pellow 2020; Peluso and Lund 2011).

### The experience of the artistic practice

The force of art as a social practice (Denis and Daniels 1988; Hawkins et al. 2015) lays on its potential to engender new worlds and alternative futures in the face of socio-ecological crisis (Hawkins et al. 2015). Nevertheless, artistic and cultural practices by local communities receive relatively little attention in environmental research. The indigenous’ Water Ceremonies during the Standing Rock protests against the Dakota Express pipeline exemplify the use of people’s cultural resources, in this case ceremonies, to resist and assert an “alternative perspective of care” (Jewett and Garavan 2019: 52). Similarly, when activists use performances in their demonstrations, including songs, drumming and dance, their bodies occupy the space and create counter-sites that enhance unique social encounters (Whatmore 2006), a kind of situated utopia, that contest and invert the real sites and propose temporary alternative forms of organization (Foucault 1984).

Examples in this line are performances by the Chilean feminist collective *Las Tesis* catalogued as prefigurative acts of solidarity and collectivity (Serafini 2020) that foster alternative ways of experiencing the public space. Similarly, the Occupy movements went beyond legal territorial strategies to generate “material practices and representations of territory” that challenged ideas of land privatization (Halvorsen 2017: 453). In fact, the potential of activist spaces of leisure, like music festivals, is such that sometimes these spaces are seen as more productive in disrupting the status quo than traditional methods of protest (Sharpe 2008). Rightly so, since leisure activities serve as a social space for individuals to organize, dialogue, and identify with one another (Hemingway 1999). These “eventful” protests sustain feelings of solidarity, agency, and the sense of community (Dellaporta 2008). Ultimately, these kind of spaces support movements and their vision of change (Prentki 2017).

Human geographers have theorized the physical and embodied experiences of participatory art-making as a place of encounter for fostering intercultural exchange (Askins and Pain 2011) as well as particular human–environment encounters (Dixon 2009; Hawkins et al. 2015). Arts-based interventions such as public murals and singing encourage connections between people, sense of community, inclusion, and dialogue (Baumann et al. 2021), and promote social cohesion and solidarity (Fancourt and Finn 2019).

The transformative potential of artistic interventions—e.g. songs, art installations, and street *performances*—does not only stem from the symbols it projects. Instead, its ephemeral and “ordinary” character disrupt the representations and forms of imperative realities (Mekdjian 2018), fostering alternative ways of inhabiting the public space. In the process, these interventions challenge the dominant hegemonic forces of privatization, thus creating conditions for political change. Yet, researchers addressing the role of creative initiatives in environmental struggles have rarely assessed art practice as a catalyst for relational values in the transformation of place, especially in degraded landscapes.

In sum, this research aims to contribute to literature on relational values, human geography and art theory by answering to the question of how engaged arts can be the pathway to restore the degraded landscape through enhanced relational values.

## Materials and methods

The gaps in the literature presented above led us to an empirical exploration of the relation between four concepts: relational values, artistic representation, artistic experience, and socio-spatial transformations. The compilation of background data started by reviewing regional press, relevant social media posts, and webpages of activist groups. The selected case study focuses on a sacrifice zone highly influenced by neoliberal policies that have been analysed by scholars from different disciplines (Bolados 2016; Espinoza et al. 2016; Tironi and Rodríguez-Giralt 2017; Valenzuela-Fuentes et al. 2021), but without focussing on the growing influence of artistic movements.

Fieldwork took place between December 2020 and March 2021. The first author of the paper conducted 35 semi-structured interviews with activists, artists, and researchers related to the QPSZ, contacted through snow-ball sampling. Interviews were conducted in-person or online (whenever interviewees preferred, in the context of the COVID-19 outbreak). Interviewees were asked to describe any artwork related to the QPSZ that they remembered and the experience, impressions, and feelings the artwork evoked. Length of the interviews varied between 30 to 90 min, and each interview was recorded and notes with observations from the interview were taken. A written consent form was obtained from all study participants in accordance with our university of affiliation’s Research Ethics Assessment. Among the interviewees, 77% were locals and 23% were external to the area, but had an activist or artistic relation with it. Regarding gender, 57% identified as women, 42% as men, and the remaining as queer or unspecified. The higher percentage of women reflect their importance in leading the mobilisations. In fact, one of the main organizations in the area, *Mujeres en Zona de Sacrificio en Resistencia* (Resistance Women in Sacrifice Zones) (MUZOSARE hereafter), actively endorses a feminist approach.

During fieldwork, a scenic art-making laboratory supplemented the data collection. The laboratory was an open-call for local activists and artists to participate in the creation of a street performance in the SZQP, organized by the first author of the paper in collaboration with the Chilean street theatre group Teatro en Movimiento Callejerx. The laboratory lasted 2 days, and 10 people participated in it. During the process, participants decided collectively the content and aim of the street performance. This activity contributed to understanding the context of the movement and the important values of the community. Observing the activation of relational values helped to define and classify them. The process was recorded, edited and shared as a short-film called “Under-ground ore” (en-tierra mineral) and shared in social media (see Sanz 2021).

A standard qualitative research data organization and analysis followed (Gallego 2005). Interviews were transcribed with Sonix and coded using MAXQDA, to capture representations and experiences of artistic practices, relational values, socio-spatial transformations, and the connections between each item that informants made during interviews. A first version of the codebook relied on secondary data, field notes, and references from the literature. This process led to the identification of 5 categories of relational values, 4 categories of artistic representations, 5 categories of experiences of art practice, and 4 categories of socio-spatial transformation used in the analysis (see the codes and definitions in Supplementary material (SM). A network analysis through Gephi 0.9.0 (and the models ForceAtlas 2, and circular layout) (Bastian et al. 2009; Jacomy et al. 2014) represents the interconnection between relational values and artistic experiences and representations. A bar chart represents the relational values activated through arts that were more influential for local transformations.

## Results and discussion

### Creative actions in the Quintero–Puchuncaví sacrifice zone

**Fig. 1:**
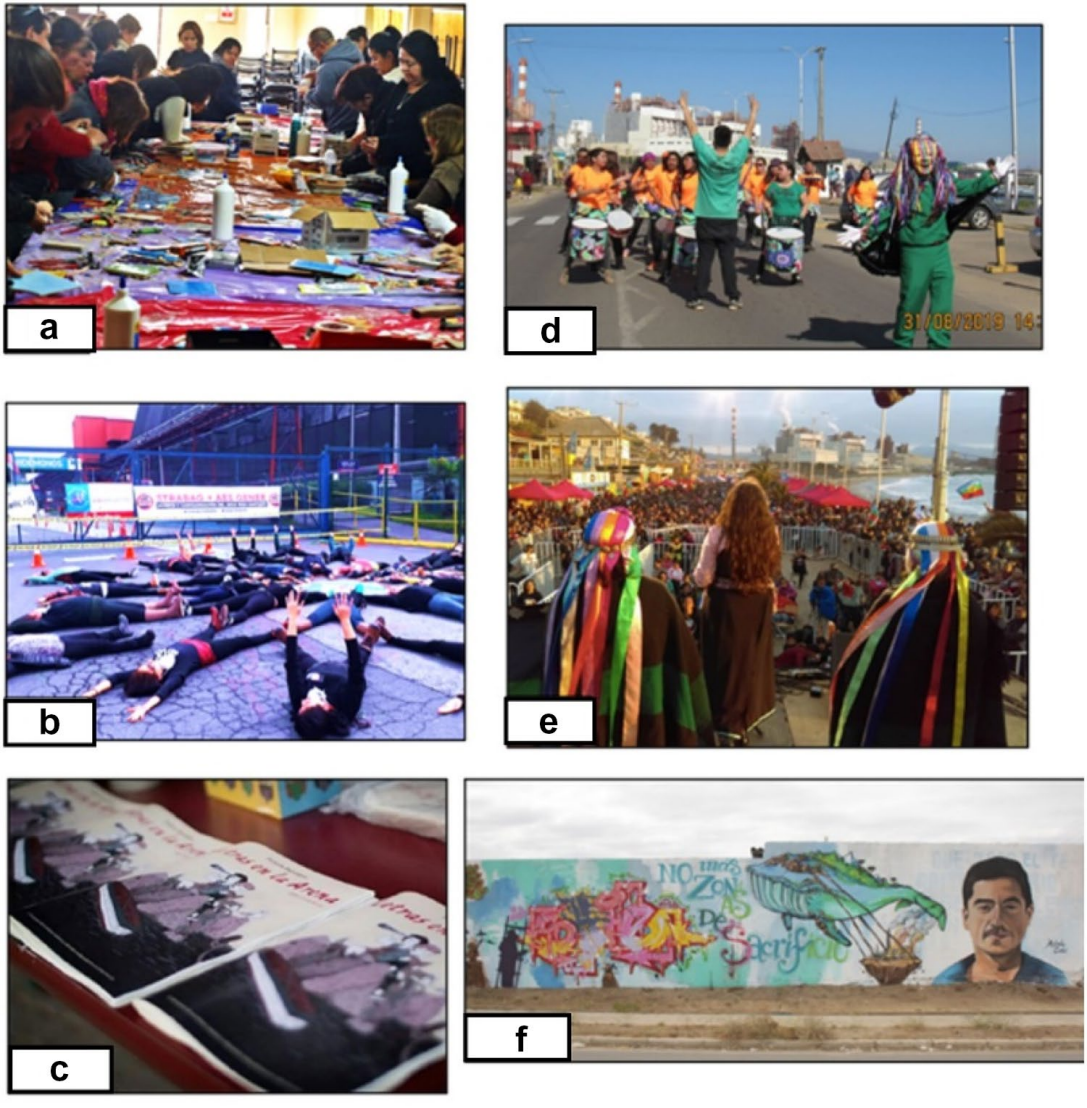
Pictures of artworks related to QPSZ. **a** Collective mosaic making Image courtesy of Efren Legazpi 2017. **b** Human mandala by MUZOSARE and MODATIMA (Image courtesy of Katta Alonso). **c** Letras en la Arena 2017 magazine. Image courtesy of Felipe Faic 2019. **d** Batuque Achelpeñ performing in Ventanas main Street, in front of the power-plant Image courtesy of Batuque Achelpeñ. **e** “Chile un solo territorio” music festival in 2018 Image courtesy of Chile un solo territorio. **f** "No mas zonas de sacrificio" mural by Murales por la Vida. Author's own photograph

When asked about artistic pieces related to the conflict, the interviewees mentioned thirty-seven creative actions across six categories used by Rodríguez-Labajos and Ray (2021) and mentioned in Table 1. The most frequent creative actions were *performances* (32%), image-based expressions (28%) and music (21%) (Fig. 1). There were fewer written expressions, such as books or fanzines, or audio-visuals. Interestingly enough, the most popular pieces, i.e. the ones that people referred to more frequently, were murals (60% of the interviewees mentioned them) and events of collective mural painting in particular (mentioned by 28.5% of the interviewees). The emblematic street art collective *Murales por la vida* (Murals for Life) (Fig. 1f) was named by 14.28% of the interviewees. Similarly, 54.3% of interviewees refer to some musical expression, in particular *Batucada*—an Afro-Brazilian percussion ensemble—(51.43%) (Fig. 1d). The music festival *Chile un solo territorio* (Chile One Territory) (Fig. 1e) named by 28.57% of the interviewees, is another emblematic event referred to around the conflict.

**Table 1:** Number of artworks according to the topic represented and location, per type of artwork

Type of artwork	Location			
	Street	Event	Multiple locations	On-line
Image based	9	1	1	
Performance-based	10	2		1
Music		5		1
Oral-written			2	
Cultural event		3		
Audio-visual	1			2

These pieces mainly occurred in the public space (43% of all pieces), although the organization of ad hoc events was also frequent (27% of all pieces) (Table 1). In particular, performative and image-based expressions tended to happen spontaneously in the public space, whereas music and cultural events took place in ad hoc events. Meanwhile, written (Fig. 1c) and audio-visual pieces were accessible online or used in multiple locations. Sixty percent of ephemeral actions involving music and *performances* were later accessible online. Interviewees also note that artistic practices—especially image-based expressions and *performances*—are often photographed and subsequently shared in social media and news stories.

### Artistic actions enhance damaged relational values in the sacrifice zone

**Fig. 2:**
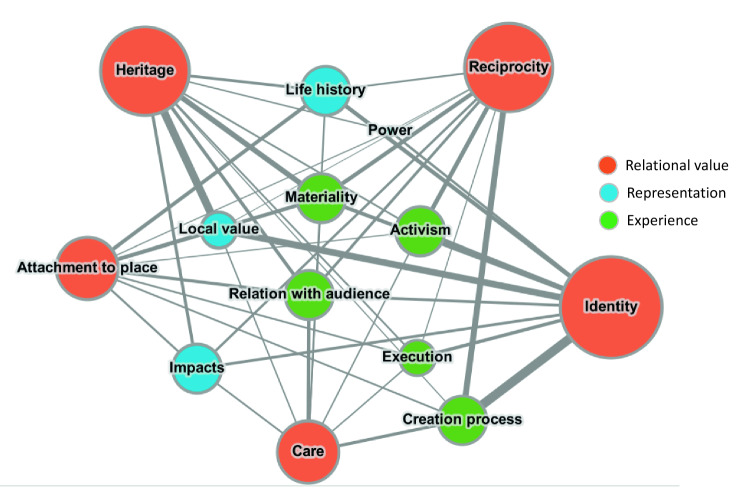
Network analysis between relational values (Orange nodes), elements and values portrayed in the pieces (blue nodes) and experience of the artistic practices (green nodes). Note: the size of the nodes and the edges reflects the number of times a given item (and their interaction) was mentioned by the interviewees. Source: Own elaboration based on force-directed Model Force Atlas 2. Since axial coding was not performed during the data organization phase, the direction of causal links was derived from the analysis of the contents but have not been represented in the network

The categories of relational values used in the analysis rely on an initial exploration of types of relations (human–human, human-place, human-time) to then propose our own terms building on the existing literature. The resulting categories, their definitions and references can be found in Table 2. Riechers et al. (2020) define relational values as meaningful human–environment connections and social relations. The interviews revealed five interconnected categories of relational values related to artistic expressions (Fig. 2), namely reciprocity, care, place attachment, identity, and heritage. The five possible categories resulted from this study do not cover all range of relational values but the ones in the intersection between artistic practices and environmental degradation.

**Table 2:** Relational values and their definition stemming from the inductive data analysis and literature review

Relational values	Definition of relational values
Reciprocity	Human–human relations (solidarity, mutual support, social cohesion, social relations) emanating from sharing spaces and interactions with the environment (Kaltenborn et al. 2017; Riechers et al. 2021)
Care	Based on Jax et al.'s (2018: 2) definitions of care as affective concern and practical actions that seek to attend to another's needs (be they other human or non-human)
Place attachment	The emotional bond between people and places, based on Manzo and Wright's (2021) “place attachment”. Place attachment is a consequence of meanings imparted on places by individuals and groups. Destruction of the landscape can drive emotions such as grief, fear, anxiety and anger derived from place attachment
Identity	Refers to the constitutive importance of environmental elements in shaping individual personalities and local culture. Based in Riechers et al.'s (2021: 5) "cultural identity" and "individual identity" and Proshansky et al.'s (1983: 60) "place identity"
Heritage	Based on ideas of cultural heritage (Kaltenborn et al. 2017) and local knowledge (Cevasco et al. 2015); appeals to the relation of people with their past, with their traditional practices, origins and history, including collective memory and local knowledge

Figure 2 shows how these relational values (orange nodes) are *represented* in the artworks (blue nodes), and *experienced* through the artistic creation (green nodes). SM provides a detailed description of the items within each category. Network analysis revealed three clusters of interactions between such representations and the experiences of relational values through arts: (1) the relevance of the creation process for identity and reciprocity; (2) the relevance of representations of local elements and life histories for heritage and place attachment, and (3) the centrality of the materiality of art in enacting a variety of relational values.

#### Activism-oriented art-making enacts identity and reciprocity

*Identity* is both the most mentioned relational value, and the best connected to artistic representation and artistic experience. Thus, the *creation process* or art-making is the best-connected artistic experience to identity. The initiative of the muralism collective *Murales por la vida* is a good example of the appeal for collective identity through participation in an art-making process. The contents of theses murals, which represent local values through images of local biodiversity, were decided collectively during creation and execution stages. Such an approach led the interviewees to assert that “Making art means socializing, building community. Making art collectively is that, creating a community” (MA, woman activist).

Likewise, *reciprocity*, that is, human–human relations that enable solidarity, mutual support and conversation, is bound with artistic practices, especially during art-making and art-for-activism events. For instance, the music festival *Chile un solo territorio* took place in front of the polluting industries to promote the motto *No más zonas de sacrificio* (no more sacrifice zones). This festival was a concrete place of encounter that provided opportunities for raising feelings of support, and a sense of community among the organizers and the participants. In other words, artistic activism enables safe spaces of affects (Ryan 2015) and especially in art-making processes, care and reciprocity is central (Fig. 1a).

These findings remind us of the notion of dialogical aesthetics developed by Kester (2004). In line with contemporary art trends, dialogical art focuses on human relationships, the interactive, and the socially-engaged experiences that art can generate. In the QPSZ, art initiatives enable new relationships and support networks between community members. When residents create an artistic piece or organize creative events for activism, art becomes a place of encounter, collective identity, and affective connections, where people feel they are part of something.

Moreover, the communication of the collective identity through art overcomes the marginalization of ideas and values that differ from the dominant vision of growth-led development. According to Bishop’s theories (2006), the purpose of participatory art is to restore the social bond through a collective elaboration of meaning. We found that artists, activists, and residents help to collectively re-build the degraded identity through creating content that portrays their aspirations and their values. Similar to our own results, Bishop spotlights the artistic experience as a source of empowerment for participants.

Political ecologists emphasize that some identities are more affected by environmental degradation than others, especially where indigenous, peasants, and racial identities are concerned (Merlinsky and Latta 2012). This is why recognition and participation of such vulnerable groups play an essential role in theories on environmental justice (Schlosberg 2007). The experience of art-making reinforces collective identity, enhances recognition, and increases participation of the marginalized identities, thus contributing to advancements of justice. Such emphasis on identity as a relational value, activated through art practices, is relatively absent from the environmental literature. A notable exception is Serafini (2018), who focuses on community arts and notes that identity is central in activism as it facilitates collective action. While we concur with this author in the link between participation in artistic projects and collective identity, the present study remarks the relevance of representation of local values and life histories in the artworks for the activation of collective identity (Fig. 2), as we discuss further in the following section.

#### Representations of local elements and life histories enact better relational values than representation of impacts

Between the relational values better enhanced by artistic practices, “heritage”, “identity”, and “place attachment*”* are directly linked with the portrayal of *local values*. A muralist explains: “When we paint the Quintero lighthouse, which is an icon of the community, they [the residents] feel identified with it; seeing it painted is very important for them because it makes them feel Quinterano, and belong to this place” (N, Local muralist).

The representation of *life histories* involves the personal experiences and interpretations of living in a sacrifice zone. Audio-visual records such as photographs, videos, and music portray the biographies and affectations of peoples living in the area. Unexpected to us, life histories play a central role in linking relational values and art (Fig. 2), as the link between relational values and art. A local activist explained: “art is a way to express the personal experiences of living here. It [art] is really personal and yet it has a tremendous collective potential. It tells the story in another way and makes people who have nothing to do with this world empathize and makes them feel identified with the feeling” (RQ, Local activist).

In the QPSZ, artistic pieces that represent local values and everyday life foster sustainable relations between people and places, thanks to the enacting of values of heritage and place attachment. On the contrary, representations of *impacts* have less impact in enhancing levering relational values (Fig. 2). This means that the visibility of an issue or impacts*, *per se, has limited transformative potential. A similar thought can be found in Demos' (2016) analysis of the aesthetics of climate change politics, where he challenges representations of impacts and catastrophic futures of climate change that silence the relations and resistance of the affected communities. In contrast, the chants about *local values and experiences* in Quintero–Puchuncaví appeal to affective bonds while unifying different voices. These chants form an example of the connection between politics and aesthetics present in philosophical discourses since the last century (Rancière 2004). This insight adds to theories on the transformative potential of ecologically engaged art (Demos 2016) but also to theories that focus on the processes and practices that maintain and disrupt relational values (Chapman et al. 2019; Peçanha Enqvist et al. 2018; West et al. 2018).

Thus, amateur and non-professional art forms are fundamental contributions in restoring human–environment relationships. Rather than just focussing on the impacts of the industries, the visualization of everyday life reconnects humans with their environment and thus fosters political changes. In this way, the discussion about pollution moves away from victimization and inaction and towards re-signification of the environment. In Quintero–Puchuncaví, when artworks represent local biodiversity, traditional economies, historical moments like pollution peaks, deaths of loving beings, and the personal experiences of deterioration of their land, culture, and history rendered invisible by the industrial development become visible. This pushes an agenda of changes towards sustainability.

#### The materiality of art as force for placemaking

Way beyond the original expectations, the *materiality* of spaces of experience and, to a lesser extent, the *relation with audiences,* are entangled with all relational values. *Materiality* refers to a specific connection and dependence to time and place of a particular piece, that is determined by the expressive medium chosen in each case. The materiality of the artistic process is made apparent by the fact that most of the pieces described by the interviewees hail from the reaction to a pollution peak in 2018. Also, as shown in Fig. 2, most of the artistic practices take place in the public space, affecting the type of relational values they stimulate. In the QPSZ, the materiality of art is a relational force as it generates spaces of encounter that enhance *reciprocity* as well as new material and symbolic spaces that challenge existing power dynamics. In the words of an interviewee: “When you do street art and do activism based on an artistic activity, more people get together than in a conventional meeting. When the *Batu* [batucada, street music performance] comes, everyone goes down to see the *Batu*. And then you can focus on making people understand that we have to fight for an area free of pollution” (M, health worker).

These results suggest that activists overcome certain barriers towards sustainability while *embodying* the creative act. Different stages of art production and materialization involve people engaged in change; people collaborating in the process of making a collective piece, interacting with an audience, occupying and re-appropriating the public space, and so on. Each one of these activities generate relational values that foster desired socio-spatial transformations. Informal artistic events in the streets of QPSZ attract people to the public space, thus enabling new relations and conversations about the injustices they are experiencing. This way, artistic events become safe and open spaces that inspire feelings of solidarity and mutual support in the face of the crisis of sense of community and privatization caused by industrial activity (Hormazábal et al. 2020).

In degraded places such as the QPSZ, large industrial facilities act as reminders of the power behind national energy interests (Broto and Calvet 2020). There, art offers a physical counterpoint to the industrial dominant landscape, through representations of resistance that explore alternative relationships with the environment. Just as the feminist performances of Las Tesis contribute to the visibility of gendered silenced bodies (Martin and Shaw 2021), when air pollution prevents certain bodies from accessing the street (Walker 2009), their mere presence in the street is an embodied act of symbolic production and power (Bourdieu 1991) and, therefore, of resistance. When activists use their bodies to produce material practices and representations of their land and values, an active praxis of embodying their right to the city leads to hope and to personal and group agency. In the words of Serafini, “what is at stake in making art in territory is the (re)making of that territory” (2022: 67). In this line, the present study proves that artistic spaces of experience in the public space transform industrialized and abandoned spaces into places of resistance and community. There, music and dancing configure care, reciprocity, memory and responsibility.

### Relational values and transformation trends

Authoritative perspectives on sustainability transformations, such as the United Nations Research Institute for Social Development (RISD) (2019: 32) acknowledge the need to address the root causes that “generate and reproduce economic, social, political, [and] environmental problems, and inequities, not merely their symptoms”. The RISD is not alone in this view. The Intergovernmental Platform on Biodiversity and Ecosystem Services (IPBES), categorizes institutions and governance systems as indirect drivers of change that influence relationships between people and nature and thus anthropogenic drivers of change, i.e. pollution (Pascual et al. 2022). Therefore, IPBES encourages the promotion of different types of values associated with the environment in decision making processes for sustainable futures (Pascual et al. 2017). This represents a great progress in the knowledge-policy interface. Despite acknowledging power relationships across stakeholders, these frameworks do not address contexts of degraded social capital or where power relations foil the construction of powerful institutions from the bottom-up and the recognition of local relational values.

**Fig. 3:**
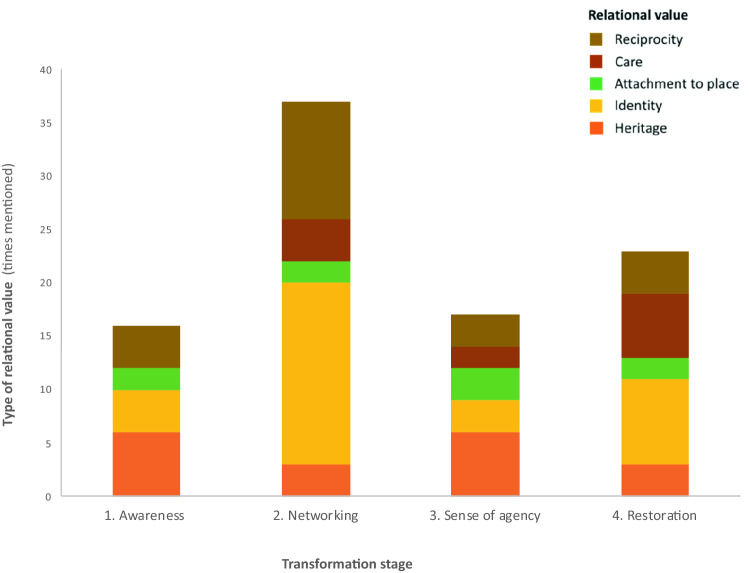
Relational values activated through arts across transformation stages in the case study

Alternative notions of transformation emphasize the transformative role of social movements. That is the case of post-development scholars such as Escobar (2004) and Svampa (2019), who show that place-based non-hierarchical networks are able to reconceptualize even the notion of development itself. This notion is especially relevant in the context addressed here, where the affected population recognize the impossibility of cancelling the industrial complex. Building on such an approach, the data from interviews revealed four stages of transformation influenced by arts in awareness, networking, sense of agency and restoration. The ultimate goal “restoration” aims to create new spaces (material and immaterial) that rehabilitate the culture, tradition, aesthetics, and places from their degraded state and also upgrade the everyday life. We accept that transformation is not necessarily a linear process. Still, Fig. 3 shows the dominant local vision about the order of the stages in the transformation, indicating the relational values activated during each transformation stage, based on the number of mentions during the interviews.

#### Creative networks towards restoration

Overall, relational values through arts have a role across all stages of transformation, especially during networking and restoration (Fig. 3). The human mandala by MUZOSARE (Fig. 1b) is an example of the intersection between networking and *identity*. "This action makes us a body, a body-territory, makes us part of something, makes us know each other, makes us communicate" (A, from MUZOSARE). This example also illustrates the relation between the values of reciprocity, identity, and care enhanced by the *experience of participating in an artistic action* (see Fig. 2). Indeed, values like care, solidary and community are central for anti-extractivist feminist discourses (Bolados and Sánchez 2017). In response to the lack of women leadership in the environmental movement, and the patriarchal and colonial system leading the QPSZ, MUZOSARE since 2016 advocates for a new ethic of care associated with Latin American feminisms (Bolados and Sánchez 2017). Unsurprisingly, the human mandala that represents *cuerpo-territorio* (body-territory) is a form of protest that MUZOSARE shares with the women’s group against hydropower in Alto Maipo river, also in Chile. The notion of *cuerpo-territorio*, popular through anti-extractivist feminist movements, acknowledges the inseparability between body and place (Bolados and Sánchez 2017). The notion comes from indigenous and decolonial ontologies of space (Zaragocin and Caretta 2020). Therefore, our results situate arts as embodied expressions of *cuerpo-territorio* and thus as avenues to decolonize spatial dynamics and analysis.

At the *restoration* stage, *identity* is again the most influential relational value, followed by *care*. Care means affective concern and actions for the community, family, and the environment. Surprisingly, *care* is the highest influence of the transformation process during restoration. This is important since identity and care are enhanced in similar experiences of art (Fig. 2), especially those focused on “safety” and “healing”. Concretely, community art-making is restorative as: “a space of healing, of dialogue, of being able to tell many things that have happened to us and feel containment. In fact, the work has been important for many women because it creates a safe space where they can share what is happening to them, and move and dance among women” (RA, performer and musician).

In this sense, our results indicate that a way to restoration goes through the transformative stages of awareness, networking, and agency. In fact, artistic processes, especially those that promote networking, can trigger individual and collective action and, ultimately, transformation. The notion of agency “emerges in crisis situations and connects networks for action” (Stockholm Resilience Centre 2009: 2).

In the QPSZ, artistic processes that emerged in situations of crisis ignited a chain of change starting with networking. Subsequently, networking activated values like reciprocity, place attachment, heritage, and identity, and in turn, promoted agency. Serafini (2018) introduced the notion of art as a medium for social and political action, giving art a prefigurative approach. Accordingly, artistic activism would be an aesthetic-political practice that fosters collective action towards social and political change. This relational notion is also present in views of art as social praxis in which communities create ephemeral, precarious, and collective ‘micro-utopias’ (Bourriaud 2004: 65). While endorsing these theories, our study establishes a co-dependence between networking and agency through relational values. Such values contribute to improve the daily life of people affected by environmental injustice and to restore their everyday landscapes. Thus, activist art can be considered as a value articulating institution (Stagl 2012) that levers the autonomy of the community and generates social cohesion, a fundamental gain when the complete rehabilitation of the environmental conditions in the sacrifice zone is far from being achieved.

## Conclusions

Sacrifice zones result from an extractive development model that obscures the exclusion of local communities’ material, symbolic, and spiritual lives, and ultimately undermines pre-existing relational values. Our work sought to trace whether (and how) these meaningful human–environment connections and social relations can be restored through art.

Local activists’ artistic practices such as banners, murals, songs, and dances have proven, through our analysis, to have relational effects that recover connections among humans, and between humans and the environment. While critical social-spatial theories acknowledge the relational nature of placemaking, previous literature had neglected the study of artistic practice as a way to repair situated relational values damaged by environmental degradation. This study has mapped the relational values activated through artistic practices that enable both representation of relational values and the experience of the values themselves. Furthermore, we propose a novel framing connecting relational values and stages of socio-spatial transformation that led to the restoration of material and symbolic spaces through art.

Our empirical evidence relies on the artistic practices developed in a sacrifice zone in the localities of Quintero and Puchuncaví, Chile, where industrial activities configure a highly toxic environment. Through participation in activism-oriented art-making projects, communities in the QPSZ are restoring the social bonds and re-building collectively the degraded community identity as they become agents of change. Representations of local elements and life histories seem to enact better relational values than representation of impacts. Making the histories and practices rendered invisible by industrial development visible, art pieces generate a critical discourse that promotes a restoration of the relation of humans with the environment.

Importantly, we identify arts’ materiality as a force of place production, as art creates material and symbolic realities that embody the right to the city. Facing intense environmental pressures, artistic spaces promote a restoration of the every-day living experiences. In this process, networking and social cohesion are essential to facilitate individual and collective agency towards transformation.

While our outcomes are context dependent, similar cases exists around the world [see EJAtlas (http://ww.ejatlas.org)]. In this context, theorizing the role of artistic practices in representing and enhancing relational values expands the toolkit for the socio-environmental restoration of degraded landscapes. These findings are an entrance point for further research on the consideration of an institutional promotion of relational values and arts-based practices in public spaces for the restoration of degraded areas.

The paper contributes to geographic research on the relational dimensions of place by expanding an understanding of the role of relational values in placemaking. Furthermore, we expand the literature on art committed to the socio-environmental crisis by pointing to the transformative dimensions of engaged arts, and on the political ecological theorization of the transformation of degraded areas, by addressing the importance of relational values and artistic practices of local communities. Above all, we aim to shed light on art activism as a powerful practice of existence/resistance in the face of current violent degradation of everyday and future environments.

## Supplementary Information

Below is the link to the electronic supplementary material.

Supplementary file1 (DOCX 83 KB)

## Data Availability

Our de-identified survey data are archived at the Dipòsit Digital de Documents de la UAB under DOI: https://ddd.uab.cat/record/259086.
