# Supplementary material for: Arts, place, and sacrifice zones: restoration of damaged relational values in a Chilean sacrifice zone
Source: Sustain Sci. 2022 Dec 15;18(3):1135–48. doi: 10.1007/s11625-022-01252-6 (PMC9753890; doi:10.1007/s11625-022-01252-6)
Supplement: Supplementary file 1 — Supplementary file1 (DOCX 83 KB) [file 11625_2022_1252_MOESM1_ESM.docx]

Supplementary material: Arts, place and sacrifice zones: restoration of damaged relational values in a Chilean Sacrifice Zone

SM1. Codebook
